# Supplementary material for: Causal relationships of Helicobacter pylori and related gastrointestinal diseases on Type 2 diabetes: Univariable and Multivariable Mendelian randomization
Source: PLoS One. 2024 Apr 23;19(4):e0300835. doi: 10.1371/journal.pone.0300835 (PMC11037534; doi:10.1371/journal.pone.0300835)
Supplement: S2 File — The original MRPRESSO result obtained by R software. (DOCX) [file pone.0300835.s002.docx]

**Results of MRPRESSO by R software**

1. **Groel to T2DM**

$`Main MR results`

Exposure MR Analysis Causal Estimate Sd T-stat P-value

1 beta.exposure Raw 0.02821355 0.01541225 1.830592 0.0719684

2 beta.exposure Outlier-corrected NA NA NA NA

1. **Groel to blood glucose levels**

$`Main MR results`

Exposure MR Analysis Causal Estimate Sd T-stat

1 beta.exposure Raw 0.01035563 0.003522018 2.940255

2 beta.exposure Outlier-corrected NA NA NA

P-value

1 0.01013206

2 NA

1. **Groel to obesity**

$`Main MR results`

Exposure MR Analysis Causal Estimate Sd T-stat

1 beta.exposure Raw -0.1073839 0.08296885 -1.294268

2 beta.exposure Outlier-corrected NA NA NA

P-value

1 0.2078886

2 NA

1. **Groel to HbA1c**

$`Main MR results`

Exposure MR Analysis Causal Estimate Sd T-stat

1 beta.exposure Raw 0.01909342 0.02002595 0.9534342

2 beta.exposure Outlier-corrected NA NA NA

P-value

1 0.3439024

2 NA

1. **Gastrointestinal disorders to T2DM**

$`Main MR results`

Exposure MR Analysis Causal Estimate Sd T-stat

1 beta.exposure Raw 0.03440694 0.01525982 2.254741

2 beta.exposure Outlier-corrected NA NA NA

P-value

1 0.02652202

2 NA

1. **Gastrointestinal disorders to obesity**

$`Main MR results`

Exposure MR Analysis Causal Estimate Sd T-stat

1 beta.exposure Raw 0.184945 0.06391674 2.89353

2 beta.exposure Outlier-corrected NA NA NA

P-value

1 0.006913439

2 NA

1. **Gastrointestinal disorders to HbA1c**

$`Main MR results`

Exposure MR Analysis Causal Estimate Sd T-stat

1 beta.exposure Raw 0.002642197 0.01791298 0.1475018

2 beta.exposure Outlier-corrected 0.012397234 0.01489692 0.8322013

P-value

1 0.8830773

2 0.4076003

1. **Gastrointestinal disorders to blood glucose levels**

$`Main MR results`

Exposure MR Analysis Causal Estimate Sd T-stat

1 beta.exposure Raw -0.003358877 0.006915494 -0.4857031

2 beta.exposure Outlier-corrected NA NA NA

P-value

1 0.6283451

2 NA

1. **Chronic gastritis to T2DM**

$`Main MR results`

Exposure MR Analysis Causal Estimate Sd T-stat

1 beta.exposure Raw 0.04203349 0.01521688 2.762293

2 beta.exposure Outlier-corrected NA NA NA

P-value

1 0.007065931

2 NA

1. **CAGA to T2DM**

$`Main MR results`

Exposure MR Analysis Causal Estimate Sd T-stat

1 beta.exposure Raw -0.001306721 0.006761648 -0.1932548

2 beta.exposure Outlier-corrected NA NA NA

P-value

1 0.8472492

2 NA

1. **Catalase to T2DM**

$`Main MR results`

Exposure MR Analysis Causal Estimate Sd T-stat

1 beta.exposure Raw -0.00152721 0.009207849 -0.1658596

2 beta.exposure Outlier-corrected NA NA NA

P-value

1 0.8687455

2 NA

1. **OMP to T2DM**

$`Main MR results`

Exposure MR Analysis Causal Estimate Sd T-stat

1 beta.exposure Raw -0.005960988 0.01181179 -0.5046642

2 beta.exposure Outlier-corrected NA NA NA

P-value

1 0.6152549

2 NA

1. **Urea to T2DM**

$`Main MR results`

Exposure MR Analysis Causal Estimate Sd T-stat

1 beta.exposure Raw -0.005012936 0.01183947 -0.4234089

2 beta.exposure Outlier-corrected NA NA NA

P-value

1 0.6731773

2 NA

1. **VacA to T2DM**

$`Main MR results`

Exposure MR Analysis Causal Estimate Sd T-stat

1 beta.exposure Raw -0.002170817 0.008294264 -0.2617251

2 beta.exposure Outlier-corrected NA NA NA

P-value

1 0.7941592

2 NA

1. **Malignant neoplasm of stomach to T2DM**

$`Main MR results`

Exposure MR Analysis Causal Estimate Sd T-stat

1 beta.exposure Raw -0.0003255332 0.005917782 -0.05500932

2 beta.exposure Outlier-corrected NA NA NA

P-value

1 0.9562834

2 NA
