# Supplementary material for: Causal relationships of Helicobacter pylori and related gastrointestinal diseases on Type 2 diabetes: Univariable and Multivariable Mendelian randomization
Source: PLoS One. 2024 Apr 23;19(4):e0300835. doi: 10.1371/journal.pone.0300835 (PMC11037534; doi:10.1371/journal.pone.0300835)
Supplement: Attachment — Submitted filename: PONE-D-23-39158_reviewer.docx [file pone.0300835.s003.docx]

**PLOS ONE**

### Causal relationships of Helicobacter pylori and related gastrointestinal diseases on Type 2 diabetes: Univariable and Multivariable Mendelian randomization

--Manuscript Draft--

| **Manuscript Number:** | PONE-D-23-39158 |
| --- | --- |
| **Article Type:** | Research Article |
| **Full Title:** | Causal relationships of Helicobacter pylori and related gastrointestinal diseases on Type 2 diabetes: Univariable and Multivariable Mendelian randomization |
| **Short Title:** | The risk of Helicobacter pylori and gastrointestinal diseases ~~on~~ in type 2 diabetes |
| **Corresponding Author:** | shouyu wang  First Affiliated Hospital of Dalian Medical University: First Affiliated Hospital of Dalian Medical University  Dalian, CHINA |
| **Keywords:** | Helicobacter pylori, gastrointestinal diseases, Type 2 diabetes mellitus, Mendelian randomization |
| **Abstract:** | Abstract  Background: Previous observational studies have demonstrated a connection between the risk of Type 2 diabetes mellitus (T2DM) and gastrointestinal problems brought on by Helicobacter pylori (H. pylori) infection. However, little is understood about how these factors impact on T2DM.  Method: This study used data from the GWAS database on H. pylori antibodies, gastroduodenal ulcers, chronic gastritis, gastric cancer, T2DM, and information on potential mediators: obesity, glycosylated hemoglobin (HbA1c), and blood glucose levels. Using univariate Mendelian randomization (MR) and multivariate MR (MVMR) analyses to evaluate the relationship between ~~of~~ H. pylori and associated gastrointestinal diseases with the ~~prevalence~~ risk of developing T2DM and explore the presence of mediators to ascertain the probable mechanisms.  **Results**: Genetic evidence suggests that H. pylori IgG antibody (P=0.006, b=0.0945, OR=1.0995, 95% CI=1.023-1.176), H. pylori GroEL antibody (P=0.028, OR=1.033, 95% CI=1.004-1.064), gastroduodenal ulcers (P=0.019, OR=1.036, 95% CI=1.006-  1.068) and chronic gastritis (P=0.005, OR=1.042, 95% CI=1.012-1.074) are all linked to an increased risk of T2DM, additionally, H. pylori IgG antibody is associated with obesity (P=0.034, OR=1.03, 95% CI=1.002-1.055). The results of MVMR showed that the pathogenic relationship between *H. pylori* GroEL antibody and gastroduodenal ulcer in T2DM is mediated by blood glucose level and obesity, respectively.  **Conclusion**: Our study found that *H. pylori* IgG antibody, *H. pylori* GroEL antibody, gastroduodenal ulcer, and chronic gastritis are all related to ~~t~~ T2DM, and blood glucose level and obesity mediate the development of *H. pylori* GroEL antibody and gastroduodenal ulcer on T2DM, respectively. These findings may inform new prevention and intervention strategies for T2DM. |
| **Order of Authors:** | Mei Sun |
|  | Zhe Zhang |
|  | Jingjing Zhang |
|  | Juewei Zhang |
|  | Zhuqiang Jia |
|  | Lin Zhao |
|  | Xin Han |
|  | Xiaohong Sun |
|  | Junwei Zong |
|  | Ying Zhu |
|  |  |

|  | Shouyu Wang |
| --- | --- |
| **Additional Information:** |  |
| **Question** | **Response** |
| **Financial Disclosure**  Enter a financial disclosure statement that describes the sources of funding for the work included in this submission. Review the [submission guidelines](http://journals.plos.org/plosone/s/submission-guidelines#loc-financial-disclosure-statement) for detailed requirements. View published research articles from [*PLOS ONE*](https://journals.plos.org/plosone/) for specific examples.  This statement is required for submission and **will appear in the published article** if the submission is accepted. Please make sure it is accurate. | Yes |
| **Funded studies**  Enter a statement with the following details:   - Initials of the authors who received each award - Grant numbers awarded to each author - The full name of each funder - URL of each funder website - Did the sponsors or funders play any role in the study design, data collection and analysis, decision to publish, or preparation of the manuscript? |  |
| Did you receive funding for this work? |  |
| Please add funding details.  as follow-up to "**Financial Disclosure**  Enter a financial disclosure statement that describes the sources of funding for the work included in this submission. Review the [submission guidelines](http://journals.plos.org/plosone/s/submission-guidelines#loc-financial-disclosure-statement) for detailed requirements. View published research articles from [*PLOS ONE*](https://journals.plos.org/plosone/) for specific examples.  This statement is required for submission and **will appear in the published article** if the submission is accepted. Please make sure it is accurate. | This study was supported by the National Natural Science Foundation of China (82074426, 82104864, 82204822), Natural Science Foundation of Liaoning Province (2021-BS-215, 2022-MS-25, 2023-MS-13), Liaoning Revitalization Talents Program (XLYC1802014), Liaoning Key Research and Development Planning Project (2017226015), Basic Research Projects of Liaoning Provincial Department of Education (LJKMZ20221286), Naural Science Foundation of Tibet Autonomous Region and Regional Science(XZ202301ZR0030G, XZ2023ZR-ZY82(Z)) and Technology Project of Naqu City. |

| **Funded studies**  Enter a statement with the following details:   - Initials of the authors who received each award - Grant numbers awarded to each author - The full name of each funder - URL of each funder website - Did the sponsors or funders play any role in the study design, data collection and analysis, decision to publish, or preparation of the manuscript? |  |
| --- | --- |
| Did you receive funding for this work?" |  |
| Please select the country of your main research funder (please select carefully as in some cases this is used in fee calculation).  as follow-up to "**Financial Disclosure**  Enter a financial disclosure statement that describes the sources of funding for the work included in this submission. Review the [submission guidelines](http://journals.plos.org/plosone/s/submission-guidelines#loc-financial-disclosure-statement) for detailed requirements. View published research articles from [*PLOS ONE*](https://journals.plos.org/plosone/) for specific examples.  This statement is required for submission and **will appear in the published article** if the submission is accepted. Please make sure it is accurate. | CHINA - CN |
| **Funded studies**  Enter a statement with the following details:   - Initials of the authors who received each award - Grant numbers awarded to each author - The full name of each funder - URL of each funder website - Did the sponsors or funders play any role in the study design, data collection and analysis, decision to publish, or preparation of the manuscript? |  |
| Did you receive funding for this work?" |  |
| **Competing Interests** | The authors have declared that no competing interests exist. |

| Use the instructions below to enter a competing interest statement for this submission. On behalf of all authors, disclose any [competing interests](http://journals.plos.org/plosone/s/competing-interests) that could be perceived to bias this work—acknowledging all financial support and any other relevant financial or non- financial competing interests.  This statement is required for submission and **will appear in the published article** if the submission is accepted. Please make sure it is accurate and that any funding sources listed in your Funding Information later in the submission form are also declared in your Financial Disclosure statement.  View published research articles from  [*PLOS ONE*](http://journals.plos.org/plosone/) for specific examples. |  |
| --- | --- |
| **NO authors have competing interests**  Enter: *The authors have declared that no competing interests exist.*  **Authors with competing interests**  Enter competing interest details beginning with this statement:  *I have read the journal's policy and the authors of this manuscript have the following competing interests: [insert competing interests here]* |  |
| * typeset |  |
| **Ethics Statement**  Enter an ethics statement for this submission. This statement is required if the study involved:   - Human participants - Human specimens or tissue - Vertebrate animals or cephalopods - Vertebrate embryos or tissues | N/A |

| - Field research   Write "N/A" if the submission does not require an ethics statement.  General guidance is provided below. Consult the [submission guidelines](http://journals.plos.org/plosone/s/submission-guidelines#loc-guidelines-for-specific-study-types) for detailed instructions. **Make sure that all information entered here is included in the Methods section of the manuscript.** |  |
| --- | --- |

| **Format for specific study types**  **Human Subject Research (involving human participants and/or tissue)**   - Give the name of the institutional review board or ethics committee that approved the study - Include the approval number and/or a statement indicating approval of this research - Indicate the form of consent obtained (written/oral) or the reason that consent was not obtained (e.g. the data were analyzed anonymously)   **Animal Research (involving vertebrate animals, embryos or tissues)**   - Provide the name of the Institutional Animal Care and Use Committee (IACUC) or other relevant ethics board that reviewed the study protocol, and indicate whether they approved this research or granted a formal waiver of ethical approval - Include an approval number if one was obtained - If the study involved *non-human primates*, add *additional details* about animal welfare and steps taken to ameliorate suffering - If anesthesia, euthanasia, or any kind of animal sacrifice is part of the study, include briefly which substances and/or methods were applied   **Field Research**  Include the following details if this study involves the collection of plant, animal, or other materials from a natural setting:   - Field permit number - Name of the institution or relevant body that granted permission |  |
| --- | --- |
|  |  |
| **Data Availability**  Authors are required to make all data underlying the findings described fully available, without restriction, and from the time of publication. PLOS allows rare exceptions to address legal and ethical concerns. See the [PLOS Data Policy](http://journals.plos.org/plosone/s/data-availability) and [FAQ](http://journals.plos.org/plosone/s/data-availability#loc-faqs-for-data-policy) for detailed information. | Yes - all data are fully available without restriction |

| A Data Availability Statement describing where the data can be found is required at submission. Your answers to this question constitute the Data Availability Statement and **will be published in the article**, if accepted. |  |
| --- | --- |
| **Important:** Stating ‘data available on request from the author’ is not sufficient. If your data are only available upon request, select ‘No’ for the first question and explain your exceptional situation in the text box. |  |
| Do the authors confirm that all data underlying the findings described in their manuscript are fully available without restriction? |  |
| **Describe where the data may be found in full sentences. If you are copying our sample text, replace any instances of XXX with the appropriate details.**   - If the data are **held or will be held in a public repository**, include URLs, accession numbers or DOIs. If this information will only be available after acceptance, indicate this by ticking the box below. For example: *All XXX files are available from the XXX database (accession number(s) XXX, XXX.*). - If the data are all contained **within the manuscript and/or Supporting Information files**, enter the following: *All relevant data are within the manuscript and its Supporting Information files.* - If neither of these applies but you are able to provide **details of access elsewhere**, with or without limitations, please do so. For example:   *Data cannot be shared publicly because of [XXX]. Data are available from the XXX Institutional Data Access / Ethics Committee (contact via XXX) for researchers who meet the criteria for access to confidential data.*  *The data underlying the results presented in the study are available from (include the name of the third party* | All phenotype data are available from the GWAS database(https://gwas.mrcieu.ac.uk). |

| *and contact information or URL).*   - This text is appropriate if the data are owned by a third party and authors do not have permission to share the data.   * typeset |  |
| --- | --- |
| Additional data availability information: |  |

Manuscript	[Click here to access/download;Manuscript;Manuscript.docx](https://www.editorialmanager.com/pone/download.aspx?id=34752382&guid=431f843c-a66c-4685-8124-8bce568367f6&scheme=1)

1 ***Causal relationships of Helicobacter pylori and related gastrointestinal diseases on Type 2***
2 ***diabetes: Univariable and Multivariable Mendelian randomization***

3	Mei Sun ^1,2¶^, Zhe Zhang ^3,4¶^, Jingjing Zhang ^5¶^, Juewei Zhang ^6¶^, Zhuqiang Jia^7,8^, Lin Zhao^9^, Xin

4 Han^8,10^, Xiaohong Sun^11^, Junwei Zong ^3*^, Ying Zhu ^1*^, Shouyu Wang ^3*^
5 ^1^Department of Infectious Diseases, the First Affiliated Hospital of Dalian Medical University,
6 Dalian, China.
7 ^2^Department of gastroenterology, Dalian Municipal Central Hospital, Dalian, China.
8 ^3^ Department of Orthopaedic Surgery, The First Affiliated Hospital of Dalian Medical University,
9 Dalian, China.
10 ^4^College of Integrative Medicine, Dalian Medical University, Dalian, China.
11 ^5^Department of Gastroenterology, the Second Affiliated Hospital of Dalian Medical University,
12 Dalian, China.
13 ^6^Health Inspection and Quarantine, College of Medical Laboratory, Dalian Medical University,
14 Dalian, China.
15 ^7^The First Affiliated Hospital of Dalian Medical University, Dalian, China
16 ^8^Naqu People's Hospital, Tibet, China.
17 ^9^Department of Quality Management, Dalian Municipal Central Hospital, Dalian, China.
18 ^10^Department of Orthopaedic Surgery, The Second Affiliated Hospital of Dalian Medical
19 University, Dalian, China.
20 ^11^Department of Nursing，The First Affiliated Hospital of Dalian Medical University, Dalian,
21 China.
22 ¶ Mei Sun, Zhe Zhang, Jingjing Zhang, Juewei Zhang have contributed equally to this work and
23 share first authorship.
24 * Corresponding author:
25 Junwei Zong: [aweizone@163.com](mailto:aweizone@163.com)
26 Ying Zhu: [zhuyingsh52@126.com](mailto:zhuyingsh52@126.com)
27 Shouyu Wang: [wangshouyu666@126.com](mailto:wangshouyu666@126.com)

28	**Abstract**

29 **Background**: Previous observational studies have demonstrated a connection between the risk of
30 Type 2 diabetes mellitus (T2DM) and gastrointestinal problems brought on by Helicobacter pylori
31 (H. pylori) infection. However, little is understood about how these factors impact on T2DM.
32 **Method**: This study used data from the GWAS database on H. pylori antibodies, gastroduodenal
33 ulcers, chronic gastritis, gastric cancer, T2DM and information on potential mediators: obesity,
34 glycosylated hemoglobin (HbA1c) and blood glucose levels. Using univariate Mendelian
35 randomization (MR) and multivariate MR (MVMR) analyses to evaluate the relationship of H.
36 pylori and associated gastrointestinal diseases with the prevalence of T2DM and exploring the
37 presence of mediators to ascertain the probable mechanisms.
38 **Results**: Genetic evidence suggests that H. pylori IgG antibody (P=0.006, b=0.0945, OR=1.0995,

39	95% CI=1.023-1.176), H. pylori GroEL antibody (P=0.028, OR=1.033, 95% CI=1.004-1.064),

40 gastroduodenal ulcers (P=0.019, OR=1.036, 95% CI=1.006-1.068) and chronic gastritis (P=0.005,
41 OR=1.042, 95% CI=1.012-1.074) are all linked to an increased risk of T2DM, additionally, H.
42 pylori IgG antibody is associated with obesity (P=0.034, OR=1.03, 95% CI=1.002-1.055). The
43 results of MVMR showed that the pathogenic relationship between Helicobacter pylori GroEL
44 antibody and gastroduodenal ulcer in T2DM is mediated by blood glucose level and obesity,
45 respectively.
46 **Conclusion:** Our study found that Helicobacter pylori IgG antibody, Helicobacter pylori GroEL
47 antibody, gastroduodenal ulcer, and chronic gastritis are all related to t T2DM~~,~~ and blood glucose
48 level and obesity mediate the development of Helicobacter pylori GroEL antibody and
49 gastroduodenal ulcer on T2DM, respectively. These findings may inform new prevention and
50 intervention strategies for T2DM.
51 **Keywords:** Helicobacter pylori, gastrointestinal diseases, Type 2 diabetes mellitus, Mendelian
52 randomization
54 H. pylori
55 Type 2 diabetes mellitus (T2DM) is a disease defined by high blood sugar levels and a relative
56 shortage of insulin, as well as insulin resistance and other chronic metabolic illnesses caused
57 predominantly by diabetes, accounting for more than 90% of diabetic patients(1). Patients with
58 T2DM have a more than 50% chance of developing complications(2), and some studies ~~have~~
59 shows that at least 80% of diabetic patients will die from cardiac complications(3, 4). Even though
60 T2DM is ~~more~~ better understood and treated than it formerly was, the disease's incidence and
61 prevalence are still rising internationally. Researchers from Sweden made the initial discovery of
62 H. pylori in a culture of human stomach mucosa(5). Around 60% of people on the planet
63 have H. pylori infections. Cell degeneration, necrosis, and inflammatory cell infiltration
64 can be caused by the inflammation and immunological response brought on by H. pylori infection,
65 and specific antibodies can be found in the serum. H. pylori is closely related to gastritis,
66 peptic ulcer, gastric cancer and other diseases(6), and *H. pylori* has been included in the
67 list of a class of carcinogens by the World Health Organization.
68 T2DM has been recognized as one of the probable illnesses of H. pylori infection in a broad
69 range of observational studies, with 61.5% of H. pylori positive patients having one or more
70 chronic diabetic sequelae(7). In T2DM patients with concurrent H. pylori infection, H. pylori
71 antibodies could be detected, and 75% of patients experienced gastrointestinal symptoms(8). H.
72 pylori infection appears to be linked to chronic inflammation, impaired insulin secretion, and
73 higher mean glycosylated hemoglobin levels, all of which increase the risk of T2DM(9, 10).
74 Additionally, it has been noted that among T2DM patients, obese people have a much greater
75 frequency of H. pylori infection than non-obese patients. However, the correlation between H.
76 pylori infection and the risk of T2DM is still debatable. According to some studies(10-12), there
77 is no distinction between diabetic and nondiabetic people in terms of the prevalence of H. pylori
78 infection. This finding may be due to sources of potential bias in observational studies, such as
79 reverse causal consistency and confounding. Therefore, stronger proof is required to show a
80 connection between H. pylori and T2DM.
81 Typical observational studies cannot demonstrate causation or rule out the impact of
82 confounding factors; they can only show whether there is a correlation between two variables. An
83 epidemiological analytic technique called Mendelian randomization (MR) makes it easier to
84 deduce causes. MR designs use single nucleotide polymorphisms (SNPs), which have a random
85 distribution without being impacted by outside influences and other confounders, as instrumental
86 variables (IVs) for target exposures(13). As a result, complex disease causal linkages can be
87 rigorously explained using MR designs. MR has been applied more frequently as a result of the
88 expansion of genome-wide association studies (GWAS) and the accessibility of massive amounts GWAS
89 data. In our study, we used seven H. pylori antibodies, as well as chronic gastritis, gastroduodenal ulcer, and
90 gastric cancer, which are closely associated with H. pylori, as exposures, and T2DM as an outcome. Then,
91 we chose obesity, glycosylated hemoglobin (HbA1c), and blood glucose levels as potential mediators to clarify
92 the association of H. pylori and its associated digestive diseases on T2DM and see whether there is a mediating
93 effect, in the hopes of elucidating the causality and offering beneficial treatment and diagnosis
94 recommendations.

# 95 Materials and Methods ~~methods~~

## 96 1. Mendelian randomized design

97 The Structure of the current MR study is shown in Figure 1, and to find the connections between
98 H. pylori and related gastrointestinal diseases and T2DM, we chose the receptors TLR1
99 (rs10004195) and FCGR2A (rs368433), which are highly correlated with H. pylori IgG antibody
100 seropositivity, as well as six H. pylori antibodies: anti-H. pylori IgG, GroEL, OMP, UREA, CagA,
101 VacA, Catalase, and the three diseases most likely to be induced by H. pylori: chronic gastritis,
102 gastroduodenal ulcer, and gastric cancer as exposure factors, obesity, HbA1c, as well as blood
103 glucose levels were selected as potential mediators, finally, T2DM was selected as an outcome to
104 elucidate the relationship between H. pylori, gastrointestinal diseases and T2DM. For the MR
105 analysis in this work, we employed genetic variants as instrumental factors. Three fundamental
106 presumptions served as the foundation for our MR study's postulated validity: (1) Correlation
107 hypothesis: genetic variation is closely related to exposure; (2) The independence hypothesis:
108 genetic variation is not associated with any confounding factors that may mediate the way from
109 exposure to outcome; (3) Exclusion-restriction hypothesis: Genetic variation can only affect the
110 result through exposure. In order to exclude the influence of heterogeneity, the random effects
111 model (IVW) was used as the main method in this study, and the MR-Egger and weighted median
112 methods were used for verification(14).
113 **Fig1:** Flowchart of Mendelian randomization analysis conducted in this study. SVMR analysis
114 investigates the effect of H. pylori infection and related gastrointestinal diseases **on T2DM development.**
115 MVMR analysis evaluates the roles of potential factors mediating the association between GroEL-
116 positive H. pylori Infection, gastroduodenal ulcer, chronic gastritis, and T2DM.

## 117 2. Data sources

118 Data for the seven antibodies to H. pylori, chronic gastritis, gastroduodenal ulcer, and diabetes
119 and potential mediators were obtained from the GWAS database (https://gwas.mrcieu.ac.uk)(15),
120 and only pooled data from European populations were used to avoid population heterogeneity bias.
121 Levels of H. pylori antibodies (GroEL, OMP, UREA, CagA , VacA and catalase), and blood
122 glucose ~~levels~~ were obtained from the dataset of the [EBI database](https://www.ebi.ac.uk/gwas/downloads/summary-statistics). ~~the data sets for~~ Gastroduodenal
123 ulcer, chronic gastritis, gastric cancer, and T2DM database were obtained from the Finnish database. ~~the~~
124 ~~data sets for~~ Glycated hemoglobin were obtained from the UK Biobank, while the obesity data was collected from GWAS summary datasets. The ~~respective~~ sample sizes were as Table 1， each GWAS was approved by the appropriate ethics committee.
125 **Table1:** Details of the studies included in the Mendelian randomization analyses.

| Phenotype | Consortium | Ethnicity | Sample size | GWAS id |
| --- | --- | --- | --- | --- |
| Helicobacter pylori CagA antibody  levels | EBI | European | 985 individuals | ebi-a-GCST90006911 |
| Helicobacter pylori Catalase antibody  levels | EBI | European | 1558 individuals | ebi-a-GCST90006912 |
| Helicobacter pylori antibody levels | EBI | European | 2716 individuals | ebi-a-GCST90006913 |
| Helicobacter pylori OMP antibody levels | EBI | European | 2640 individuals | ebi-a-GCST90006914 |
| Helicobacter pylori UREA antibody  levels | EBI | European | 2251 individuals | ebi-a-GCST90006915 |
| Helicobacter pylori antibody levels | EBI | European | 1571 individuals | ebi-a-GCST90006916 |
| Gastroduodenal ulcer | FinnGen | European | 4510 cases, 189,695, controls | finn-b-K11_GASTRODUOULC |
| Chronic gastritis | FinnGen | European | 5,213 cases, 189,695 controls | finn-b-K11_CHRONGASTR |
| Malignant neoplasm of stomach | FinnGen | European | 633 cases, 218,159 controls | finn-b-CD2_BENIGN_STOMACH |
| Obesity | GIANT | European | 2896 cases, 47,468 controls | ieu-a-92 |
| HbA1c | UK Biobank | European | 13,586,180 SNPs | ukb-d-30750_raw |
| Blood glucose levels | EBI | European | 400,458 individuals | ebi-a-GCST90025986 |
| Type 2 diabetes | FinnGen | European | 24,133 cases, 183,185 controls | finn-b-E4_DM2NASCOMP |

## 126 3.Selection of potential genetic variants ~~instrumental variables~~

127 Genetic variants can be obtained in two ways, either directly from GWAS summary statistics
128 or through existing reports in the literature. In this study, genes strongly associated with H. pylori
129 infection were selected ~~for study~~ based on previously available reports and confirming that existing
130 studies have chosen this approach for H. pylori-associated Mendelian studies (16, 17). The SNPs
131 rs10004195 at TLR1 (4p14) and rs368433 at FCGR 2A gene (1q23.3) have been identified as
132 genetic variants associated with high H. pylori seropositivity, ~~which could be extracted~~
133 ~~from H. pylori IgG antibodies~~(18). To check the significance of the allelic score as a tool, the F-
134 statistics of both SNPs were calculated to be greater than 10 (Table 2). Other data on H. pylori
135 antibodies, gastrointestinal diseases, T2DM, and potential mediators were obtained from GWAS
136 summary statistics. A variety of tests are necessary to screen for genetic IVs that are eligible and
137 satisfy the MR hypothesis. First, to boost statistical efficacy and get a sufficient number of IVs,
138 we set the p-value threshold for IVs in this MR study to 5E-05 to screen for SNPs that were
139 associated with exposure and strongly associated with exposure. Second, independence was set to
140 eliminate linkage disequilibrium (LD: r^2^ = 0.001, kb = 10,000, p < 5E-05) and the statistical
141 significance was calculated (F-statistics). The F-statistics greater than 10 indicates the absence of
142 weak instrumental variable bias (8). Third, to ensure that the effect alleles are members of the same
143 allele, coordinate the exposure and outcome data sets. These exacting processes can be utilized to
144 screen SNPS, which can then be used as IV for further examination.
145 **Table 2:** Instrumental SNPs of IgG-positive H. pylori Infection and F statistics.

| SNP | Beta | SE | EA | NEA | EAF | Pval | F-statistics |
| --- | --- | --- | --- | --- | --- | --- | --- |
| rs10004195 | 0.3576744 | 0.04048331 | A | T | 0.25 | 1.00E-18 | 78.059 |
| rs368433 | 0.3148107 | 0.05609599 | C | T | 0.16 | 2.00E-08 | 31.495 |

## 146 4. Statistical analysis and data visualization

147 TwoSampleMR, MR-PRESSO, MVMR, and forestploter packages in R software are used for
148 analysis, and inverse variance weighting (IVW) is used as the default method to evaluate the causal
149 estimate(19). For MR Analysis with more than two IVS, two complementary methods MR-Egger
150 and weighted median are used for verification. A P-value less than 0.05 was considered statistically
151 significant. To ensure the reliability of the results, we further carried out a sensitivity analysis,
152 that is, heterogeneity and Pleiotropyt test. A P-value less than 0.05 indicates the existence of
153 heterogeneity and pleiotropy. Then, the MR-Egger intercept test is used to evaluate the validity of
154 the hypothesis of instrumental variables. The leave-one-out method is used to analyze whether
155 there are SNPS that drive the results independently, and the MR-PRESSO test is used to evaluate
156 whether there are outlier SNPS. For MVMR, we adjusted for T2DM as an outcome and for glucose
157 levels and obesity, respectively, to explore whether they mediated the causal relationship between
158 anti-H. pylori GroEL and gastroduodenal ulcer, respectively, and T2DM.

161	**Results**

162 The number of SNPs screened as instrumental variables significantly associated with exposure
163 through LD ranged from 38 to 444 (S1 File)), and the F-statistic for each SNP included in the study
164 was greater than 10 (Table 3).
165 **Table3:** The number of SNPs and F-statistics of Phenotype.

| Phenotype | F statistics | nSNP |
| --- | --- | --- |
| Helicobacter pylori CagA antibody levels | 19.613 | 90 |
| Helicobacter pylori Catalase antibody levels | 18.523 | 80 |
| Helicobacter pylori GroEL antibody levels | 18.039 | 68 |
| Helicobacter pylori OMP antibody levels | 18.605 | 86 |
| Helicobacter pylori UREA antibody levels | 18.691 | 88 |
| Helicobacter pylori VacA antibody levels | 18.704 | 93 |
| obesity | 21.959 | 38 |
| HbA1c | 53.192 | 444 |
| blood glucose | 61.164 | 271 |
| Gastroduodenal ulcer | 19.044 | 92 |
| Chronic gastritis | 18.578 | 84 |
| Malignant neoplasm of stomach | 18.693 | 73 |

## 166 Univariate Mendelian randomization

### 167 1. Relationship between potential mediators and T2DM

168 The relationship between obesity, HbA1c, blood glucose level and T2DM has been validated in
169 many experiments, and to ensure the feasibility of the Mendelian randomization study, we used
170 the validation by IVW method with genetically predicted obesity (P=0.023, OR=1.069,

171	95%CI=1.008-1.133), HbA1c (P=1.263E-76, OR=1.233, 95% CI=1.206-1.261), and blood

172 glucose levels (P=8.21E-42, OR=4.077, 95% CI=3.327-4.996) were significantly associated with
173 an increased risk of T2DM. To validate this relationship, we then used the MR Egger method and
174 Weighted median, and the results remained significant (obesity: MR Egger: P=0.023, OR=1.254,

175	95% CI=1.040-1.512; Weighted median: P=0.00002, OR=1.080 95% CI=1.042-1.119; blood

176	glucose level: MR Egger: P=6.60E-14, OR=4.331, 95% CI=3.013-6.225; Weighted median:

177	P=1.99E-20, OR=2.916, 95% CI=2.325-3.656; HbA1c : MR Egger: P=5.011E-16, OR=1.195, 95%

178	CI=1.147-1.246; Weighted median: P=3.5E-20, OR=1.130, 95% CI=1.101- 1.159). In the

179 sensitivity analysis results showed heterogeneity in all three, but no pleiotropy, and none of the
180 egger intercepts significantly deviated from 0. Therefore, obesity, HbA1c, and blood glucose
181 levels can be used as potential mediators for Mendelian randomization analysis (Fig 2).
182 **Fig2:** Mendelian randomization results of the effect of potential mediators (obesity, HbA1c, blood
183 glucose level) on T2DM.

### 184 2. Effect of Helicobacter pylori antibodies on T2DM and its potential mediators

185 According to the IVW method, genetically predicted anti-H. pylori igG was substantially linked

186	to a higher risk of T2DM (P=0.006, b=0.0945, OR=1.0995, 95% CI=1.023-1.176), as well as

187	obesity (P=0.034, OR=1.03, 95% CI=1.002-1.055), but the results with HbA1c (P=0.105,

188 OR=0.930, 95% CI=0.852 to 1.015) were not significant enough for causality, and the test for
189 heterogeneity showed that there was no heterogeneity in these results, which was insufficient for
190 other tests and multivariate analyses due to the limitation of the number of snp. Anti-H. pylori
191 GroEL was significantly in connection with an elevated risk of T2DM by the IVW method
192 (P=0.028, OR=1.033, 95% CI=1.004-1.064), in accordance with the results obtained by the MR Egger (P=0.04, OR=1.07,
194 95% CI=1.003-1.143) and Weighted median (P=0.004, OR=1.06, 95% CI=1.018-1.096). ~~and~~ ~~the results of~~ The
195 sensitivity analysis showed the presence of heterogeneity, the absence of Pleiotropy (Fig 3), and
196 none of the Egger intercepts significantly deviated from 0. The MR-PRESSO results showed the absence
197 of an outlier SNP (S2 File). In addition, anti-H. pylori GroEL was associated with increased blood glucose
198 levels (Fig 4), in agreement with ~~which was verified in~~ IVW (P=0.020, OR=1.011, 95% CI=1.002-1.021 ) and
199 Weighted median (P=0.029, OR=1.014, 95% CI=1.001- 1.027 ) methods. In contrast, it was not significant in MR
200 Egger method (P=0.111 , OR=1.017, 95% CI=0.997- 1.038). Concerning the sensitivity analysis, the
201 results showed no Heterogeneity and pleiotropy, none of the egger intercepts significantly deviated from 0,
202 and the MR-PRESSO results also showed the absence of outlying SNP (S2 File)). In addition, ~~to~~ the leave-one-
203 out method of alignment analysis showed the absence of a single SNP causes the causal consequences of anti-
204 H. pylori GroEL on T2DM and blood glucose levels (Fig 5). However, anti-H. pylori GroEL was not
199 causally associated with obesity and HbA1c, and none of the other antibody- H. pylori were causally
200 associated with T2DM.
201 **Fig3:** Mendelian randomization results of the effect of Helicobacter pylori antibodies,
202 Gastroduodenal ulcer, Chronic gastritis, Malignant neoplasm of stomach on T2DM.
203 **Fig4:** Mendelian randomization results of the effect of Helicobacter pylori antibodies,
204 gastroduodenal ulcer, chronic gastritis on potential mediators (obesity, HbA1c, blood glucose
205 level).
206 **Fig5:** (A-B) Mendelian randomization result of the leave-one-out method of anti-H.pylori GroEL
207 on T2DM and blood glucose levels.
208 (C-D) Mendelian randomization result of the leave-one-out method of gastroduodenal ulcer on
209 T2DM and obesity.
210 (E) Mendelian randomization results of the leave-one-out method of chronic gastritis on T2DM.

### 211 3. Impact of gastrointestinal disorders on T2DM and potential intermediaries

212 Genetically predicted gastroduodenal ulcer and chronic gastritis were both in connection with
213 an elevated risk of T2DM, nevertheless, there was no causal relationship between Malignant neoplasm of
214 stomach and T2DM (Fig 3). By using the IVW method, gastroduodenal ulcer was found to be
215 significantly associated with an increased risk of T2DM (P=0.019, OR=1.036, 95% CI=1.006-
216 1.068), but not relevant when utilizing the weighted median（P=0.410, OR=1.016，95%CI=0.978-
217 1.055) or MR-Egger (P=0.071, OR=1.056，95%CI=0.996-1.120). ~~This was followed by tests for~~
218 The assessment of the heterogeneity and pleiotropy ~~which~~ revealed the presence of heterogeneity and
219 the absence of pleiotropy. ~~that~~ The Egger intercept did not significantly deviate from 0, the leave-one-out
220 permutation analyses revealed the lack of a single SNP with a causal effect on T2DM, the MR-PRESSO
221 results also revealed the absence of outlier SNPs (S2 File)). Furthermore, the gastroduodenal ulcer was also
222 linked to an increased risk of obesity as determined by the IVW method (P=0.009, OR=1.204, 95%
223 CI=1.047 to 1.385), but was not significant in the other two methods, there was no heterogeneity
224 and pleiotropy (Fig 4), and the leave-one-out method showed the absence of individual SNPs driving
225 the causal effect on obesity (Fig 5). ~~while~~ According to the results, the gastroduodenal ulcer was not causally
226 associated with HbA1c and blood glucose levels.
227 Regarding the chronic gastritis lesions, the results showed a significant link between chronic gastritis and an increased risk of T2DM (P=0.005, OR=1.042, 95% CI=1.012-1.074), and the ~~same~~ result agreed with
228 ~~obtained in~~ the weighted median approach (P=0.042, OR=1.046, 95% CI=1.002-1.092). While in MR Egger
229 the result was ~~were~~ not significant, sensitivity analysis showed no heterogeneity or pleiotropy, and the
230 Egger intercept was almost close to 0, the leave-one-out method also showed that there was no SNP
231 that individually drives a causal effect on T2DM(Fig 5), MR-PRESSO results showed that there
232 was no standing group of SNP (S2 File). ~~but there was~~ No causal relationship between chronic
233 gastritis and any of the three candidate mediators was revealed (Fig 4)

## 234 Multivariate Mendelian randomization

### 235 1. Mediating relationship between anti-H. pylori GroEL and blood glucose

236 **levels with T2DM**
237 MVMR was carried out to determine whether blood glucose levels were a mediating factor in
238 the connection between GroEL antibody-positive H. pylori and T2DM. When blood glucose levels
239 were adjusted, the association between T2DM and anti-H. pylori GroEL disappeared (P = 0.131, OR

245	= 1.085, 95% CI = 0.976-1.205) in the IVW method, but the link between blood glucose levels and

246 the disease ~~was~~ still persisted (P = 3.82E-41, OR = 4.071, 95% CI = 3.317-4.996). The results of
247 MVMR using Lasso methods showed consistent results with the IVW method (Fig 6).
248 **Fig6:** Multivariable MR result of causal relationships of anti-H.pylori GroEL and blood glucose
249 levels on T2DM.
250 **2.Mediating relationship between Gastroduodenal ulcer and obesity with T2DM**
251 The relationship between T2DM and obesity-mediated gastroduodenal ulcer was examined
252 using MVMR. The ~~MVMR~~ results revealed that while the relationship between gastroduodenal
253 ulcer and T2DM disappeared after combining the instrumental variables of obesity and
254 gastroduodenal ulcer, the association between obesity and T2DM remained substantial (P = 0.541,
255 OR = 1.033, 95% CI = 0.931-1.146). Similarly, the MVMR results of Lasso were consistent with
256 IVW method (Fig 7).
257 **Fig7:** Multivariable MR result of causal relationships of gastroduodenal ulcer and obesity on
258 T2DM.

259	**Discussion**

260 Although evidence indicates that H. pylori infection is linked to a higher likelihood of T2DM,
261 the exact cause and underlying mechanisms remain unknown. We investigated the causal
262 relationships among seven different types of H. pylori antibodies, gastroduodenal ulcer,
263 chronic gastritis, gastric cancer, and T2DM by using univariate Mendelian randomization,
264 and we investigated whether these causal relationships were mediated using multivariate
265 Mendelian randomization. ~~Finally, we came to the conclusion~~ The results showed that the relationship among
266 anti-H. pylori IgG, anti-H. pylori Groel, gastroduodenal ulcer, chronic gastritis and type 2 diabetes is
267 causally related. In addition, fasting glucose and obesity, respectively, act as mediators in the
268 relationships between anti-H. pylori Groel, gastroduodenal ulcer, and TD2M.
269 Antibodies anti- H. pylori are found in the infected patients ~~with H. pylori infection~~. It is reported, that a
270 higher levels of antibodies anti-H. pylori is linked to T2DM ~~in the past~~ (20). ~~and~~ An observational
271 study found that more than 75% of diabetic patients had elevated H. pylori antibodies and suffered from
272 gastric problems (9). SNP rs 10004195 of the Toll-like receptor 1 (TLR 1) gene at 4p 14 and SNP rs368433 of
273 the FCGR A gene at 1Q 23.3 have been identified as genetic variants with the strongest association for H.
274 pylori sero-positive rates(18). TLR1's A allele has been linked to an increase in H. pylori infections, and FCGR
275 polymorphisms have been linked to a number of persistent bacterial infections(17, 21).
276 By analyzing the original literature, it was discovered that these two receptors were taken from H.
277 pylori serum igG antibodies. These two receptors were studied by Mendelian randomization in
278 prior relevant studies on behalf of H. pylori as an exposure factor(16, 22). Mendelian study of
279 these two receptors with T2DM and potential mediators revealed a strong association between
280 them and an elevated risk of both T2DM and obesity. However, due to the limited number of SNPs,
281 it was not sufficient to carry out the next step of the multivariate Mendelian randomization study;
282 thus, we reasonably hypothesized by combining previous reports in the literature and our findings
283 that obesity may be a mediating factor in the increase of T2DM by anti-H. pylori igG, and our
284 speculation is consistent with a study on the correlation of H. pylori and obesity with T2DM, which
285 indicated that the prevalence of H. pylori infection is increased in obese patients with T2DM, and
286 obese patients with T2DM had a greater rate of H. pylori infection(8).
287 GroEL is a chaperone protein that is necessary for the proper folding of several proteins in
288 bacteria(23). Previous research has revealed that GroEL is a novel H. pylori virulence factor that
289 is extensively expressed in most H. pylori strains and may act as a possible indicator for high-risk
290 H. pylori infections. Antibodies against GroEL are linked to H. pylori infections and are capable
291 of sticking to gastric epithelial cells and triggering inflammatory responses(24), which is an
292 independent predictor of gastric lesion progression(25). Furthermore, GroEL is cross-reactive in
293 humans and H. pylori, and this cross-reactive antibody may contribute to the inflammatory
294 response, raising the risk of T2DM(26). Our MR study suggest that GroEL antibodies not only
295 increase the risk of T2DM, but also raise blood glucose levels, which is similar with the findings
296 of Ningning You et al. on H. pylori and blood glucose(27), who demonstrated that H. pylori
297 infection is a distinct risk factor for elevated blood glucose levels in nondiabetic individuals, and
298 that persistent H. pylori infection leads to elevated fasting plasma glucose and TG/HDL levels,
299 which give rise to the development of T2DM. This could be because H. pylori infection increases
300 the consumption of a high-fat diet, which alters the diversity of key intestinal bacteria, causing an
301 imbalance in intestinal homeostasis, increasing lipid metabolism, and ultimately leading to an
302 imbalance in glucose metabolism(28), and previous research has shown that intestinal
303 microbiology plays a significant role in the hyperglycemia brought on by H. pylori(29). Therefore,
304 it is imperative to get eradicated of H. pylori infections as soon as they are found in order to prevent
305 T2DM and blood glucose abnormalities from developing as a result of chronic infections.
306 The role of Helicobacter pylori infection in peptic ulcer, gastric cancer, gastritis, and other
307 gastrointestinal disorders is becoming more and more clearly recognized with the passage of
308 time(30). In an observational study, as many as 75% of diabetic patients responded with significant
309 gastrointestinal symptoms(31, 32), presenting with dysphagia, reflux, constipation, stomach
310 discomfort, nausea, vomiting, and diarrhea(7). It has been reported that the release of gastric-
311 related hormones may be influenced by H. pylori-induced gastritis, and among these hormones,
312 leptin, hunger hormone, gastrin, and growth inhibitor all affect the susceptibility to diabetes and
313 promote obesity and diabetes(33, 34). The following are some potential mechanisms at play: High
314 levels of leptin may hamper function of pancreatic islet, inhibit insulin production in response to
315 glucose and cause human pancreatic B-cell death via activating c-JNK(35); gastrin increases food-
316 related and glucose-stimulated insulin release(36), growth inhibitor controls pancreatic insulin
317 secretion and prevents insulin release, while starvation hormone lowers energy expenditure and
318 encourages weight gain(37). In our study, we carried out a Mendelian randomization study of the
319 three diseases most likely to be induced by H. pylori with T2DM, and the results showed that
320 gastric cancer was not causally related to T2DM, while gastroduodenal ulcer and chronic gastritis
321 were associated with the development of T2DM, and in order to further explore the mechanism of
322 the occurrence of this causality, we compared the two diseases with the T2DM development,
323 respectively. Mendelian analysis of obesity, HbA1c and blood glucose levels, respectively, which
324 showed that gastroduodenal ulcer was a risk factor for increased risk of obesity, so we
325 hypothesized that obesity might be a mediator between gastroduodenal ulcer and T2DM , and then
326 we carried out the validation, and the result was in line with our speculation that gastroduodenal
327 ulcer mediated T2DM	through obesity, which was in line with the results of previous results of
328 a Mendelian study on the association between peptic ulcer and obesity (38), therefore, obese
329 patients with gastroduodenal ulcer should be carefully managed to prevent T2DM.
330 In conclusion, among the exposures adopted in this study, anti-H. pylori IgG, GroEL,
331 gastroduodenal ulcer and chronic gastritis were causally associated with T2DM, which suggests a
332 close relevance between gastrointestinal disorders and the condition. Patients with H. pylori should
333 pay close attention to T2DM-related indicators to achieve early detection and prompt treatment.
334 Additionally, this study and other article reports highlight the crucial relationship between obesity
335 and T2DM, thus people with H. pylori infection must pay attention to weight control to stop the
336 condition from progressing.
337 The strengths of this study are the selection of data from larger studies, the full inclusion of
338 indicators of H. pylori infection and associated gastrointestinal disorders as exposures, the first
339 exploration of the association between H. pylori and T2DM by Mendelian randomization, and the
340 use of multivariate MR analyses to explore mediating pathways in order to identify a number of
341 possible mechanisms, filling a gap in the randomized controlled trials. There are also some
342 limitations of this study. First, to obtain sufficient instrumental variables, the P-value threshold
343 chosen for IV was 5E-05, which may introduce a weak instrumental bias to the overall estimates.
344 Second, there is a small dataset on H. pylori infection in the GWAS database, and although we
345 used snps and antibodies strongly associated with it to do the study and get positive results, there
346 is still a need for more comprehensive data to line up a broader study on H. pylori. Third, in order
347 to avoid the bias of population heterogeneity, we only based the GWAS summary statistics on the
348 population of European descent, and the applicability of these results to other ethnic groups needs
349 to be further explored.

350	**Conclusion**

351 Our research discovered that H. pylori IgG antibody, H. pylori GroEL antibody, gastroduodenal
352 ulcer, and chronic gastritis are all linked to type 2 diabetes. Additionally, blood glucose levels and
353 obesity serve as intermediaries between H. pylori GroEL antibody, gastroduodenal ulcerand type
354 2 diabetes, respectively. This study indicates that Helicobacter pylori eradication therapy can
355 decrease the risk of developing type 2 diabetes. Furthermore, controlling blood sugar and weight
356 can help reduce the risk of developing type 2 diabetes in individuals with H. pylori infection and
357 gastroduodenal ulcer.

# 358 Acknowledgements

359 We gratefully acknowledge the authors and participants of all GWASs from which
360 we used summary statistics data.

# 361 Consent for publication

362	Not applicable.

# 363 Availability of data and materials

364 The data utilized in this research was publicly accessible and could be downloaded from the
365 following online platforms or repositories.

# 366 Ethics approval and consent to participate

367 The data used in this study were all from public databases that can be downloaded directly for
368 research purposes and do not involve the reporting or using of any animal, human, or tissue data.

# 369 conflict of interest statement

370	There is no conflict of interest.

371	**Funding**

372 This study was supported by the National Natural Science Foundation of China (82074426,
373 82104864, 82204822), Natural Science Foundation of Liaoning Province (2021-BS-215, 2022-
374 MS-25, 2023-MS-13), Liaoning Revitalization Talents Program (XLYC1802014), Liaoning Key
375 Research and Development Planning Project (2017226015), Basic Research Projects of Liaoning
376 Provincial Department of Education (LJKMZ20221286), Naural Science Foundation of Tibet
377 Autonomous Region and Regional Science(XZ202301ZR0030G, XZ2023ZR-ZY82(Z)) and
378 Technology Project of Naqu City.

379	**Reference**

380 1.	Chatterjee S, Khunti K, Davies MJ. Type 2 diabetes. The lancet. 2017;389(10085):2239-51.
381 2.	He C, Yang Z, Lu N-H. Helicobacter pylori infection and diabetes: is it a myth or fact? World
382 Journal of Gastroenterology: WJG. 2014;20(16):4607.
383 3.	Agardh E, Allebeck P, Hallqvist J, Moradi T, Sidorchuk A. Type 2 diabetes incidence and
384 socio-economic position: a systematic review and meta-analysis. International journal of

385	epidemiology. 2011;40(3):804-18.

386 4.	Qi L, Hu FB, Hu G. Genes, environment, and interactions in prevention of type 2 diabetes: a
387 focus on physical activity and lifestyle changes. Current molecular medicine. 2008;8(6):519-32.
388 5.	Malfertheiner P, Megraud F, O’Morain C, Bazzoli F, El-Omar E, Graham D, et al. Current
389 concepts in the management of Helicobacter pylori infection: the Maastricht III Consensus Report.

390	Gut. 2007;56(6):772-81.

391 6.	Liu WZ, Xie Y, Lu H, Cheng H, Zeng ZR, Zhou LY, et al. Fifth Chinese National Consensus
392 Report on the management of Helicobacter pylori infection. Helicobacter. 2018;23(2):e12475.
393 7.	Bakka AS, Salih BA. Prevalence of Helicobacter pylori infection in asymptomatic subjects in
394 Libya. Diagnostic microbiology and infectious disease. 2002;43(4):265-8.
395 8.	Bener A, Micallef R, Afifi M, Derbala M, Al-Mulla HM, Usmani MA. Association between
396 type 2 diabetes mellitus and Helicobacter pylori infection. Turk J Gastroenterol. 2007;18(4):225-.
397 9.	Nodoushan SAH, Nabavi A. The interaction of Helicobacter pylori infection and type 2
398 diabetes mellitus. Advanced Biomedical Research. 2019;8.
399 10. Kouitcheu Mabeku LB, Noundjeu Ngamga ML, Leundji H. Helicobacter pylori infection, a
400 risk factor for Type 2 diabetes mellitus: a hospital-based cross-sectional study among dyspeptic
401 patients in Douala-Cameroon. Scientific reports. 2020;10(1):12141.
402 11. Zhou F, Zhong X, Chen J, Li C, Shang M, Jiang C, et al. Helicobacter pylori infection
403 associated with type 2 diabetic nephropathy in patients with dyspeptic symptoms. Diabetes
404 research and clinical practice. 2015;110(3):328-34.
405 12. Sotuneh N, Hosseini SR, Shokri-Shirvani J, Bijani A, Ghadimi R. Helicobacter pylori
406 infection and metabolic parameters: is there an association in elderly population? International
407 journal of preventive medicine. 2014;5(12):1537.
408 13. Cheng L, Zhuang H, Yang S, Jiang H, Wang S, Zhang J. Exposing the causal effect of C-
409 reactive protein on the risk of type 2 diabetes mellitus: a Mendelian randomization study. Frontiers

410	in genetics. 2018;9:657.

411	14. Lawlor DA, Harbord RM, Sterne JA, Timpson N, Davey Smith G. Mendelian randomization:

412	using genes as instruments for making causal inferences in epidemiology. Statistics in medicine.

413	2008;27(8):1133-63.

414	15. Lyon MS, Andrews SJ, Elsworth B, Gaunt TR, Hemani G, Marcora E. The variant call format

415	provides efficient and	robust storage of GWAS summary statistics. Genome biology.

416	2021;22(1):32.

417 16. Liu Y, Xu H, Zhao Z, Dong Y, Wang X, Niu J. No evidence for a causal link between
418 Helicobacter pylori infection and nonalcoholic fatty liver disease: A bidirectional Mendelian
419 randomization study. Frontiers in Microbiology. 2022;13:1018322.
420 17. Tang F-b, Li Z-x, Wang Y-m, Zhang L, Ma J-l, Zhou T, et al. Toll-like receptor 1 and 10
421 polymorphisms, Helicobacter pylori susceptibility and risk of gastric lesions in a high-risk Chinese
422 population. Infection, Genetics and Evolution. 2015;31:263-9.
423 18. Mayerle J, den Hoed CM, Schurmann C, Stolk L, Homuth G, Peters MJ, et al. Identification
424 of genetic loci associated with Helicobacter pylori serologic status. Jama. 2013;309(18):1912-20.
425 19. Hemani G, Zheng J, Elsworth B, Wade KH, Haberland V, Baird D, et al. The MR-Base
426 platform supports systematic causal inference across the human phenome. elife. 2018;7:e34408.
427 20. Feldman R. EPIDEMIOLOGIC OBSERVATIONS AND OPEN QUESTIONS ABOUT
428 DISEASE AND INFECTION CAUSED BY HELICOBACTER PYLORI. ACHTMAN M,
429 SERBAUM S HELICOBACTER PYLORI: MOLECULAR AND CELLULAR BIOLOGY. 2001.
430 21. Corcoran PA, Byrne MF. Is the host responsible?–the role of the immune system in
431 Helicobacter pylori infection. Medical hypotheses. 2004;62(1):95-6.
432 22. Li B, Zhang Y, Zheng Y, Cai H. Causal Effect of Helicobacter Pylori Infection on Coronary
433 Heart Disease is mediated by Body Mass Index: A Mendelian Randomization Study. medRxiv.

434	2023:2023.03. 08.23287014.

435	23. Dunn B, Roop 2nd R, Sung CC, Sharma S, Perez-Perez G, Blaser M. Identification and

436	purification of a cpn60 heat shock protein homolog from Helicobacter pylori. Infection and

437	Immunity. 1992;60(5):1946-51.

438	24. Guo X, Tang P, Zhang X, Li R. Causal associations of circulating Helicobacter pylori

439	antibodies with stroke and the mediating role of inflammation. Inflammation Research. 2023:1-10.

440	25. Pan KF, Formichella L, Zhang L, Zhang Y, Ma JL, Li ZX, et al. Helicobacter pylori antibody

441	responses and evolution of precancerous gastric lesions in a Chinese population. International

442	journal of cancer. 2014;134(9):2118-25.

443	26. Yamaguchi H, Osaki T, Kai M, Taguchi H, Kamiya S. Immune response against a cross-

444	reactive epitope on the heat shock protein 60 homologue of Helicobacter pylori. Infection and

445	immunity. 2000;68(6):3448-54.

446 27. You N, Chen Y, Yan X, Gu B, Wang J, Zhang J. Persistent Helicobacter pylori infection leads
447 to elevated fasting plasma glucose level: A retrospective cohort study based on a nondiabetes
448 Chinese population. Journal of Gastroenterology and Hepatology. 2023.
449 28. Moreira A, Texeira T, Ferreira A. Peluzio Mdo C, Alfenas Rde C. Influence of a high-fat diet
450 on	gut	microbiota,	intestinal	permeability	and	metabolic	endotoxaemia.	Br	J	Nutr.

451	2012;108(5):801-9.

452	29. Bonfigli A, Boemi M, Festa R, Bonazzi P, Brandoni G, Spazzafumo L, et al. Randomized,

453	double-blind, placebo-controlled trial to evaluate the effect of Helicobacter pylori eradication on

454	glucose homeostasis in type 2 diabetic patients. Nutrition, Metabolism and Cardiovascular

455	Diseases. 2016;26(10):893-8.

456 30. Hosseini E, Poursina F, Van de Wiele T, Safaei HG, Adibi P. Helicobacter pylori in Iran: A
457 systematic review on the association of genotypes and gastroduodenal diseases. Journal of research
458 in medical sciences: the official journal of Isfahan University of Medical Sciences. 2012;17(3):280.
459 31. Granquist Å, Bredberg A, Sveger T, Axelsson I. A longitudinal cohort study on the prevalence
460 of Helicobacter pylori antibodies in Swedish children and adolescents. Acta Paediatrica.

461	2002;91(6):636-40.

462 32. Rajashekhar V, Bhasin D, Ray P, Vaiphei K, Sharma B, Singh K. Helicobacter pylori infection
463 in chronic smokers with non ulcer dyspepsia. Tropical Gastroenterology: Official Journal of the
464 Digestive Diseases Foundation. 2000;21(2):71-2.
465 33. Jeffery PL, McGuckin MA, Linden SK. Endocrine impact of Helicobacter pylori: focus on
466 ghrelin and ghrelin o-acyltransferase. World journal of gastroenterology: WJG. 2011;17(10):1249.
467 34. Roper J, Francois F, Shue PL, Mourad MS, Pei Z, Olivares de Perez AZ, et al. Leptin and
468 ghrelin in relation to Helicobacter pylori status in adult males. The Journal of Clinical
469 Endocrinology & Metabolism. 2008;93(6):2350-7.
470 35. Maedler K, Schulthess FT, Bielman C, Berney T, Bonny C, Prentki M, et al. Glucose and
471 leptin induce apoptosis in human β‐ cells and impair glucose‐ stimulated insulin secretion through
472 activation of c‐ Jun N‐ terminal kinases. The FASEB Journal. 2008;22(6):1905-13.
473 36. Peach H, Barnett N. Helicobacter pylori infection and fasting plasma glucose concentration.
474 Journal of clinical pathology. 2001;54(6):466-9.
475 37. Tschöp M, Smiley DL, Heiman ML. Ghrelin induces adiposity in rodents. Nature.

476	2000;407(6806):908-13.

477	38. Li Z, Chen H, Chen T. Genetic liability to obesity and peptic ulcer disease: a Mendelian

478	randomization study. BMC Medical Genomics. 2022;15(1):209.

# 479 Supporting information

480	**S1 File. SNPS information for tool variables.** SNPS for Mendelian randomization analysis after

481	linkage imbalance and adjustment of p-values for all instrumental variables

482	**S2 File. Results of MRPRESSO by R software.** The original MRPRESSO result obtained by R

483	software.

Fig1	[Click here to access/download;Figure;Fig1.tif](https://www.editorialmanager.com/pone/download.aspx?id=34737455&guid=96a07a72-3242-4920-857e-439ac5f5dd79&scheme=1)

Fig2	[Click here to access/download;Figure;Fig2.tif](https://www.editorialmanager.com/pone/download.aspx?id=34737459&guid=0b4b6b03-7d46-497e-b7c0-ae82820cd3ae&scheme=1)

Fig3	[Click here to access/download;Figure;Fig3.tif](https://www.editorialmanager.com/pone/download.aspx?id=34737463&guid=afbb3916-d3f5-49c9-ab3a-b2a7818d2dec&scheme=1)

Fig4	[Click here to access/download;Figure;Fig4.tif](https://www.editorialmanager.com/pone/download.aspx?id=34737465&guid=aa7b3c75-9140-4f76-86ce-d84c9a46cb7d&scheme=1)

Fig5	[Click here to access/download;Figure;Fig5.tif](https://www.editorialmanager.com/pone/download.aspx?id=34737474&guid=f1b827ef-e96c-41fc-9efa-76ce412cc01a&scheme=1)

Fig6	[Click here to access/download;Figure;Fig6.tif](https://www.editorialmanager.com/pone/download.aspx?id=34737477&guid=ffe4f2e5-128f-4cb1-ae48-8fbe2785eb8a&scheme=1)

Fig7	[Click here to access/download;Figure;Fig7.tif](https://www.editorialmanager.com/pone/download.aspx?id=34737479&guid=9c74d98a-a356-499d-bf0c-f3dea2a04afd&scheme=1)

S2 FIle

[Click here to access/download](https://www.editorialmanager.com/pone/download.aspx?id=34737609&guid=caad7880-b675-44f2-ac97-2f96fe986152&scheme=1)

[**Supporting Information** S2 FIle.docx](https://www.editorialmanager.com/pone/download.aspx?id=34737609&guid=caad7880-b675-44f2-ac97-2f96fe986152&scheme=1)

S1 File

[Click here to access/download](https://www.editorialmanager.com/pone/download.aspx?id=34737542&guid=2619ea19-83bb-4cd0-80aa-d10fd7d992ac&scheme=1)

# [Supporting Information - Compressed/ZIP File Archive S1 File.zip](https://www.editorialmanager.com/pone/download.aspx?id=34737542&guid=2619ea19-83bb-4cd0-80aa-d10fd7d992ac&scheme=1)
